# 

**Published:** 1882-05

**Authors:** 


### CONTENTS.

### COMMUNICATIONS.

Nature's Royal Family........................................................ 207
The Relation of Dentistry to Medicine........................................ 219
Dental Pathology and Surgery................................................. 226
The Disorders of Primary Dentition........................................... 231
The Cause and Prevention of Dental Caries.................................... 237
Operative Dentistry.......................................................... 242
Hypodermic Administration of Carbolic Acid................................... 246

### CORRESPONDENCE.

From E. G. B.................................................................. 249

### PROCEEDINGS.

Baltimore College of Dental Surgery, 251.—Boston Dental College, 251. —Ohio College of Dental Surgery, 252.—Philadelphia Dental College, 252.—Pennsylvania College of Dental Surgery, 253.—New York College of Dentistry, 253.—Missouri Dental College, 254.—Western College of Dental Surgeons, 254.—University of Michigan—College of Dental Surgery, 254.—Dental Department of the University of Tennessee, 255.—Indiana Dental College, 255.—Dental Department of Vanderbilt University, 255.—University of Pennsylvania—Dental Department, 256.

### EDITORIAL.

An Important Step............................................................. 256
American Dental Association.................................................. 258
Illinois State Dental Society................................................. 259
Northern Ohio Dental Association.............................................. 260

